# Necroptosis is a key mediator of enterocytes loss in intestinal ischaemia/reperfusion injury

**DOI:** 10.1111/jcmm.12987

**Published:** 2016-09-28

**Authors:** Shihong Wen, Yihong Ling, Wenjing Yang, Jiantong Shen, Cai Li, Wentao Deng, Weifeng Liu, Kexuan Liu

**Affiliations:** ^1^ Department of AnesthesiologyNanfang HospitalSouthern Medical UniversityGuangzhouChina; ^2^ Department of AnesthesiologyThe First Affiliated HospitalSun Yat‐sen UniversityGuangzhouChina; ^3^ Collaborative Innovation Center for Cancer MedicineState Key Laboratory of Oncology in South ChinaSun Yat‐sen University Cancer CenterGuangzhouChina; ^4^ Department of PathologySun Yat‐sen University Cancer CenterGuangzhouChina; ^5^ Department of AnesthesiologyThe First Affiliated HosptialZhengzhou UniversityZhengzhouChina

**Keywords:** intestine, ischaemia/reperfusion injury, programmed necrosis, high‐mobility group box‐1

## Abstract

Cell death is an important biological process that is believed to have a central role in intestinal ischaemia/reperfusion (I/R) injury. While the apoptosis inhibition is pivotal in preventing intestinal I/R, how necrotic cell death is regulated remains unknown. Necroptosis represents a newly discovered form of programmed cell death that combines the features of both apoptosis and necrosis, and it has been implicated in the development of a range of inflammatory diseases. Here, we show that receptor‐interacting protein 1/3 (RIP1/3) kinase and mixed lineage kinase domain‐like protein recruitment mediates necroptosis in a rat model of ischaemic intestinal injury *in vivo*. Furthermore, necroptosis was specifically blocked by the RIP1 kinase inhibitor necrostatin‐1. In addition, the combined treatment of necrostatin‐1 and the pan‐caspase inhibitor Z‐VAD acted synergistically to protect against intestinal I/R injury, and these two pathways can be converted to one another when one is inhibited. *In vitro*, necrostatin‐1 pre‐treatment reduced the necroptotic death of oxygen‐glucose deprivation challenged intestinal epithelial cell‐6 cells, which in turn dampened the production of pro‐inflammatory cytokines (tumour necrosis factor‐α and interleukin‐1β), and suppressed high‐mobility group box‐1 (HMGB1) translocation from the nucleus to the cytoplasm and the subsequent release of HMGB1 into the supernatant, thus decreasing the activation of Toll‐like receptor 4 and the receptor for advanced glycation end products. Collectively, our study reveals a robust RIP1/RIP3‐dependent necroptosis pathway in intestinal I/R‐induced intestinal injury *in vivo* and *in vitro* and suggests that the HMGB1 signalling is highly involved in this process, making it a novel therapeutic target for acute ischaemic intestinal injury.

## Introduction

Ischaemic injury to the gut frequently occurs in acute mesenteric ischaemia, traumatic or septic shock, and certain operative procedures including small bowel transplantation and abdominal aortic surgery 1. Intestinal ischaemia and subsequent reperfusion (I/R) is associated with local and systemic injuries that ultimately progress to multiple organ dysfunction syndrome and often have an overall mortality as high as 60–80% 2, 3. To date, a limited number of pharmacological agents have been shown to provide some benefit in intestinal I/R injury conditions, although without complete success 4.

Intestinal I/R induces the generation of excessive cell death (*i.e*. apoptosis and necrosis), which are the major causes of mucosal epithelial barrier dysfunction. We and other researchers previously demonstrated that a key aspect of intestinal I/R injury is the increased occurrence of apoptotic cell death in the intestine 5, 6, 7, 8. Recent insight into the process of cell death by apoptosis and necrosis in intestinal I/R injury models has begun to shed light on the role of these pathways in the process of injury and subsequent repair 9, 10, 11. Apoptosis has long been considered as the best‐known form of programmed cell death. When excessive, however, it may impair the epithelial barrier, leading to severe gut pathology 12, 13. Unlike apoptosis, necrosis is described as an uncontrolled and accidental form of cell death until genetically determined, regulated processes that mediates necrotic cellular demise, which were recently identified and are now termed programmed necrosis or necroptosis 14, 15. Necroptosis shares with necrosis the fact that dying cells display the morphological features of necrosis but not of apoptosis, but is highly regulated by an intracellular protein platform 14, 16. Recent advances have shown that activation of the kinase domain of receptor‐interacting protein1 (RIP1) and the assembly of RIP1/3‐containing signalling complex (termed the necrosome) mediated necroptosis contributes to the pathogenesis in preclinical models of brain 17, 18, heart 19, 20, and kidney 21, 22 I/R injury, which can be protected by using the RIP1 kinase inhibitor necrostatin (Nec)‐1. Meanwhile, the necrosome phosphorylates the mixed lineage kinase domain‐like protein (MLKL), which subsequently results in the rapid, active, and dynamic release of cell damage‐associated molecular patterns (DAMPs) following the loss of plasma membrane integrity and promotes ongoing inflammation and secondary tissue injury 23. High‐mobility group protein B1 (HMGB1) is a typical DAMP molecule because intracellular HMGB1 is present in all nucleated cells and is critical in the homoeostasis of most living cells, whereas extracellular HMGB1 can initiate and sustain the inflammatory response through ligation of pattern recognition receptors, including receptor for advanced glycation endproducts and toll‐like receptors 24.

With the recognition of necroptosis, an intriguing opportunity to unravell the molecular events and potential pharmacological interference avenues for therapeutic intervention has emerged. In addition, there is emerging evidence that the necroptotic signalling pathway has a general role in the modulation of intestinal inflammatory disorders, including inflammatory bowel disease and ulcerative colitis 25, 26. As such, it has become clear that at least some part of regulated necrotic cell death in the gut is pathological. However, it remains unclear whether programmed necrosis is present in the pathophysiological course of acute ischaemic intestinal injury.

Herein, we aimed to demonstrate the functional relevance of necroptosis in regulating intestinal I/R injury *in vivo* and oxygen‐glucose deprivation (OGD)‐challenged intestinal epithelial cell (IEC‐6) injury *in vitro*. In addition, we identified that the simultaneous inhibition of necroptosis and apoptosis using Nec‐1 and Z‐Val‐Ala‐Asp(OMe)‐fluoromethyl ketone (Z‐VAD, an irreversible apoptosis inhibitor) during intestinal I/R confers enhanced intestinal protection and served as a novel prophylactic target for intestinal injury.

## Materials and methods

### Animal model and treatment groups

All animal protocols were approved by the National Animal Care and Use Committee of Sun Yat‐sen University.

Adult male Sprague‐Dawley rats weighing 220–250 g were anesthetized with pentobarbital (30 mg/kg, intraperitoneally). The model of intestinal I/R was established as previously reported 27. During the study period, body temperature was maintained at 37°C with the aid of a heating blanket. After reperfusion, the resuscitation was carried out by administering intraperitoneally 0.5 ml/100 g of normal saline and the wound was then closed by sterile suture.

To evaluate the functional relevance of RIP1‐mediated necroptotic cell death in *in vivo* model, rats were randomly assigned to the following groups: (*i*) sham group in which animals just underwent isolation of the superior mesenteric artery, but without occlusion; (*ii*) I/R group in which rats received 1 hr of ischaemia followed by reperfusion; and (*iii*) Nec‐1 group in which rats received Nec‐1 (1.0 mg/kg, dissolved in normal saline, intraperitoneally) 22 30 min. before ischaemia and other manipulations for I/R animals. The above cohort of rats (*n* = 8 per group) received 6 hrs of reperfusion. Another cohort of rats (sham group, I/R group and Nec‐1 group, *n* = 8 per group) received identical procedures as described above for each group but followed by 24 hrs of reperfusion.

The third cohort of rats was randomized into six groups (*n* = 8 per group): (*i*) sham group; (*ii*) I/R group; (*iii*) Nec‐1 group (1.0 mg/kg, dissolved in normal saline, intraperitoneally); (*iv*) irreversible pan‐caspase inhibitor Z‐VAD group [ALX‐260‐020; Enzo Lifescience (Farmingdale, NY, USA), 1.5 mg/kg, intraperitoneally] 28; (*v*) dimethylsulfoxide (DMSO) group, in which DMSO was the solvent of Z‐VAD and served as the vehicle control and (*vi*) Nec‐1 + Z‐VAD combined treatment group. The above drugs were administered 30 min. before ischaemia and the animals underwent 1 hr of ischaemia followed by 24 hrs of reperfusion.

### Preparation of specimens

After euthanizing the rats, blood samples were taken from ventricle and centrifuged, and the supernatant was stored at −80°C for subsequent measurements of diamine oxidase (DAO) activity and tumour necrosis factor (TNF)‐α levels. A 5–10 cm segment of the intestine was further divided into three segments. The segments were, respectively, fixed in 10% neutral formaldehyde and paraffin embedded for morphological analysis; snap‐frozen in liquid nitrogen immediately and cut into frozen sections for immunofluorescence staining; or washed with cold saline, scraped, dried with filter paper and preserved at −80°C.

### Histological measurement of intestinal mucosal injury

Paraffin sections (4 μm thickness) of the small intestinal segments were prepared and stained with haematoxylin‐eosin. The degree of injury was evaluated using the Chiu's score method as previously described 29.

### *In vitro* cell culture

Intestinal epithelial cells were obtained from American Type Culture Collection (Cat. RL‐1592; Manassas, VA, USA) and cultured in DMEM containing 10% FBS, 4.5 g/l d‐glucose and 1% penicillin/streptomycin. The cells were incubated at 37°C in a humidified atmosphere with 5% CO_2_ and allowed to reach 70–80% confluence before each passage.

### Oxygen and glucose deprivation model and cell treatments

To simulate intestinal I/R *in vivo*, IEC‐6 cell injury was induced by an OGD procedure as reported previously 30. Briefly, the culture medium was replaced by warm glucose‐free Earle's balanced salt solution (EBBS; ionic composition in mM: NaCl 116.36, NaHCO_3_ 26.18, NaH_2_PO_4_ 1.00, KCl 5.36, CaCl_2_ 1.8, MgSO_4_ 0.8; pH 7.4) bubbled through with pure nitrogen gas for 15 min. Then, the cells treated with or without necroptosis and apoptosis inhibitors in multi‐well plates were placed in an airtight chamber (AnaeroLab system, Labmed, Guangzhou, China) equipped with vacuum air pump and inflator, and flushed by a gas mixture consisting of 95% N_2_ and 5% CO_2_. Control cells were incubated in EBBS containing 5 mM glucose in a normoxic incubator. After OGD treatment, cells were removed from the gas chamber, the EBBS in the OGD‐exposed and control cells were replaced with pre‐warmed culture medium and incubated for up to 24 hrs under normoxic conditions (reoxygenation). Cell viability was assessed using an 3‐(4,5‐Dimethyl‐2‐thiazolyl)‐2,5‐diphenyl‐2H‐ tetrazolium bromide (MTT, 5 mg/ml in PBS, Sigma‐Aldrich, St. Louis, MO, USA) assay.

The cells were divided into six groups (*n* = 6 per group): (*i*) control group cells were maintained in culture medium; (*ii*) OGD group; (*iii*) DMSO group; (*iv*) Z‐VAD group cells were pre‐treated with Z‐VAD (10 μM); (*v*) Nec‐1 group cells were pre‐treated with Nec‐1 (20 μM) and (*vi*) Nec‐1 + Z‐VAD group cells were pre‐treated combined with Nec‐1 (20 μM) and Z‐VAD (10 μM). All the inhibitors were administrated 1 hr before OGD. DMSO was the solvent of Z‐VAD and at the concentrations used was without any effect on cell viability.

### Quantification of DAO, TNF‐α and HMGB1

Diamine oxidase, a sensitive marker reflecting small intestinal mucosal injury 31, was detected using a chemical assay kit (Nanjing Jiancheng Biochemicals Ltd., Nanjing, China) according to the protocol. Tumour necrosis factor‐α level in the serum were measured with an ELISA kit (Boster, Wuhan, China) in accordance with the instructions. High‐mobility group protein B1 (HMGB1) concentrations in the cell culture supernatants were measured using a HMGB1 ELISA kit (Shino‐Test Corporation, Sagamihara, Japan) according to the manufacturer's instructions.

### Immunofluorescence staining

For the *in vivo* analysis, frozen intestines were sliced into 8 μm thick sections for immunofluorescence staining according to standard procedures. Briefly, sections were rinsed in 4% paraformaldehyde (PFA) for 10 min. at room temperature (RT), and then washed three times in PBS‐Tween 20 (PBST, 0.1% Triton X‐100 in PBS, pH 7.4). Next, the sections were incubated with 3% goat serum diluted in PBS for 1 hr at RT, and were then incubated with anti‐TNF‐α receptor 1 (TNFR1; ab19139; Abcam, Cambridge, MA, USA) or anti‐cleaved caspase 3 (9664; Cell Signaling Technology, Shanghai, China) in diluting buffer (3% bovine serum albumin, 0.1% in PBS) overnight at 4°C. To study HMGB1 translocation *in vitro*, cells were fixed in PFA in 0.1% PBS solution. The cells were incubated in 10% normal donkey serum in 0.1% PBST and were then incubated overnight with anti‐HMGB1 (ab18256; Abcam). After rinsing in PBS, the intestine sections and cells were incubated at RT for 1 hr with secondary antibodies conjugated with Alexa Fluor^®^ 488 (ab150077; Abcam) or Alexa Fluor^®^ 555 (ab150074; Abcam). For double labelling with cleaved caspase‐3, terminal deoxynucleotidyl transferase mediated dUTP‐biotin nick end labelling (TUNEL) staining was applied to a series of sections and cells using the DeadEnd^™^ Fluorometric TUNEL System (G3250; Promega, Fitchburg, WI, USA) according to the manufacturer's suggestions. After that, Hoechst 33258 (H1399; Invitrogen, Carlsbad, CA, USA) was used to stain the nuclei (blue). The slides were imaged with an inverted fluorescence microscope (Observer Z1; Carl Zeiss, Thornwood, NY). Immunofluorescence was quantified using Image J (National Institutes of Health, Bethesda, MD, USA) and the background was subtracted. Ten representative regions per section (*in vivo*) or field (*in vitro*) were randomly selected by an assessor blinded to the treatment groups.

### Quantitative real‐time PCR

Total RNA was extracted from IEC‐6 cells using Trizol reagent (Invitrogen). cDNA was synthesized using SuperScript II (Invitrogen). Real‐time PCR analysis was performed with the PrimeScript^™^RT reagent Kit and the SYBR Premix EX Taq II Kit (TaKaRa, Kyoto, Japan) on a C1000 thermal cycler and the ABI7300 real‐time PCR system (Applied Biosystems, Foster City, CA, USA). The following primer pairs were used: F: 5′‐CCAAATGGGCTCCCTCTCAT‐3′, R: 5′‐TCCGCTTGGTGGTTTGCTAC‐3′ for TNF‐α; F: 5′‐GCAGCATCTCGACAAGAGCTT‐3′, R: 5′‐GCTCCACGGGCAAGACATAG‐3′ for interleukin (IL)‐1β, F: 5′‐GTATGACTCTACCCACGGCAAGT‐3′, and R: 5′‐TCTCGCTCCTGGAAGATGGT‐3′ for GAPDH. Relative mRNA quantification was performed with the ΔΔCt method and GAPDH was used as the endogenous standard for each sample. For graphical representation, the ΔΔCt values were normalized to controls and expressed as the fold change.

### Immunoprecipitation and Western blot analysis

Protein was extracted from intestinal mucosal samples and IEC‐6 cells. Cytoplasmic protein extraction was performed with a commercially available kit (Beyotime, Beijing, China). For RIP1 immunoprecipitation, 2 mg protein were pre‐cleared with protein G‐Sepharose beads for 2 hrs and incubated with the RIP1 antibody or control IgG O/N at 4°C. For western blot, total protein was electrophoresed on 10% or 12% SDS‐PAGE and transferred to PVDF membrane. After blocking, the blots were further incubated overnight 4°C with cleaved Caspase‐3 (9661; Cell Signaling Technology), RIP1 (610459; BD Biosciences, Franklin Lakes, NJ, USA), RIP3 (ADI‐905‐242; Enzo Lifescience), MLKL (sc‐165025; Santa Cruz, Dallas, TX, USA), HMGB1 (ab18256; Abcam), Toll‐like receptor 4 (TLR4, sc‐10741; Santa Cruz), Receptor for advanced glycation end products (RAGE, sc‐5563; Santa Cruz). The membranes were washed and incubated with the secondary antibodies (IRDye 800CW Goat antimouse/Rabbit IgG, Licor‐biosciences, Lincoln, NE, USA). After additional washing, the blots were analysed by the LICOR Odyssey infrared imaging system. β‐actin (4970; Cell Signaling Technology) served as the internal control, and the results were expressed as a ratio relative to β‐actin.

### Statistical analysis

SPSS 13.0 software (SPSS Inc., Chicago, IL, USA) was used for data analyses. Data are expressed as mean ± S.D. and analysed by one‐way anova followed by the Tukey *post hoc* procedure for multiple comparisons. *P* < 0.05 was considered statistically significant.

## Results

### Necrostatin‐1 protects against intestinal tissue injury after ischaemia/reperfusion

**Figure 1:**
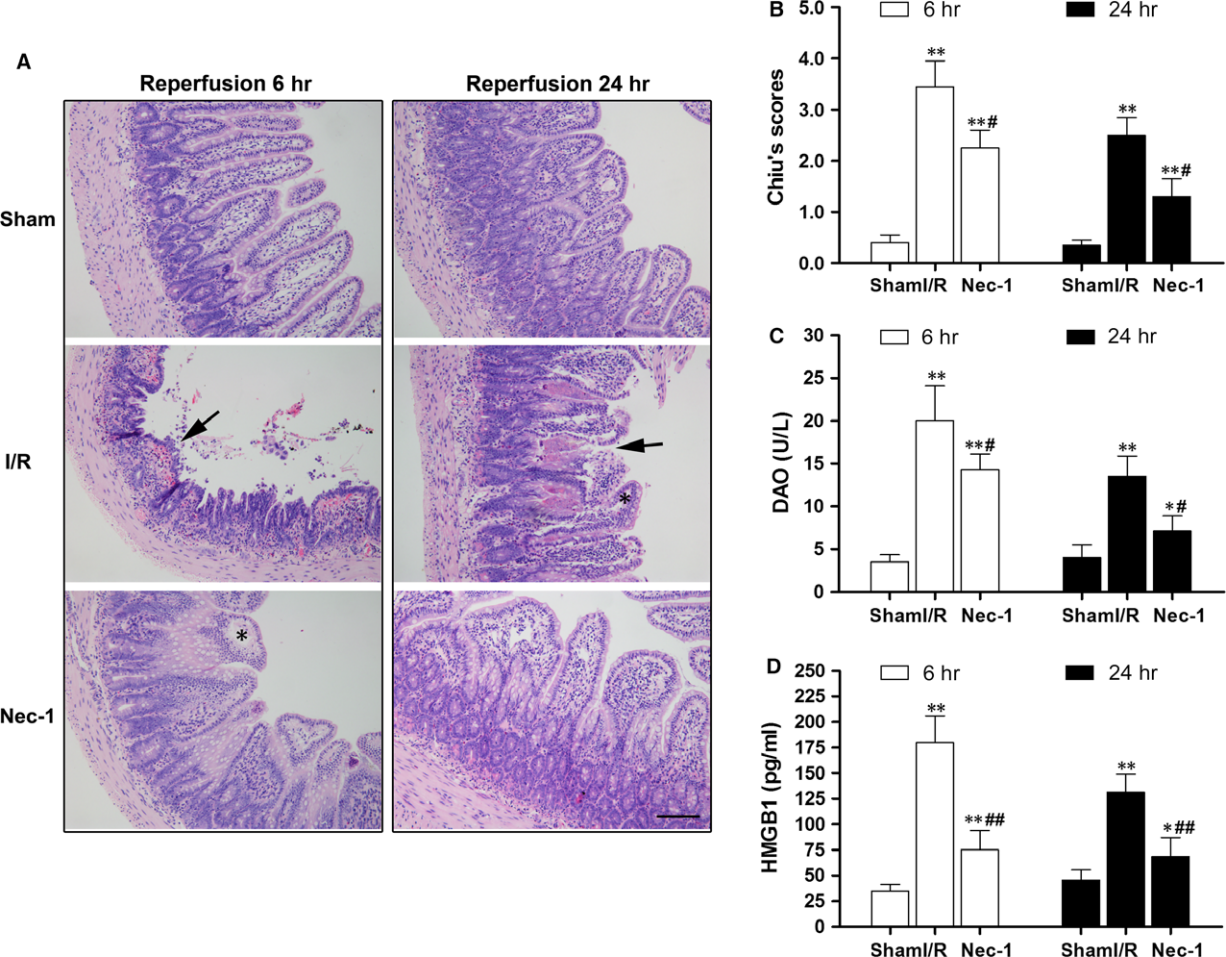
Intestinal protection by necrostatin‐1 *versus* intestinal I/R injury *in vivo*. (**A**) Histopathologic changes in the intestinal mucosa. Haematoxylin and eosin stained small intestine after 1 hr ischaemia followed by 6 hrs/24 hrs of reperfusion. Magnification is ×200, bar denotes 100 mm. Black arrows indicate denuded, fused villi and haemorrhage. Black asterisks indicate Gruenhagen's space. (**B**) Injury scores of the intestinal mucosa morphology. (**C**) Intestinal cellular injury evaluated by serum DAO activity. (**D**) Serum HMGB1 level were analysed by ELISA. The images are representative for each group. The data are shown as the means ± S.D. (*n* = 8 per group). *P < 0.05, **P < 0.01 compared with sham group, ^#^ *P* < 0.05, ^##^ *P* < 0.05 compared with I/R group.

Representative intestine sections are shown in Figure 1A. The intestine of the sham rat exhibited normal mucosal architecture. Typical hallmarks of severe intestinal I/R injury, such as destruction and loss of epithelial cells, infiltration of inflammatory cells, dramatic reduction in the villi and dilated capillaries appeared in the I/R‐treated group. In contrast, there was relative preservation of the mucosal structure in the Nec‐1 treated groups. In parallel with the mucosal morphologic changes, results of the evaluation of the intestinal damage scores are shown in Figure 1B. A marked reduction in intestinal tissue damage was achieved by a single application of Nec‐1 (all *P* < 0.05) when compared with the I/R groups after 6 and 24 hrs reperfusion.

Sham‐operated rats that were followed up for 6 and 24 hrs of reperfusion had basal serum DAO levels of 3.5 ± 0.8 U/l and 4.0 ± 1.2 U/l, respectively. As expected, I/R‐treated rats had increased serum levels of DAO at 6 and 24 hrs after reperfusion. A statistically significant reduction in DAO levels was detected after Nec‐1 administration (Fig. 1C, *P* < 0.05). We also detected a high level of serum HMGB1 after I/R as compared to sham rats, and Nec‐1 administration reduced the extracellular HMGB1 levels (Fig. 1D).

### Necroptosis is present during the process of intestinal ischaemia/reperfusion injury

**Figure 2:**
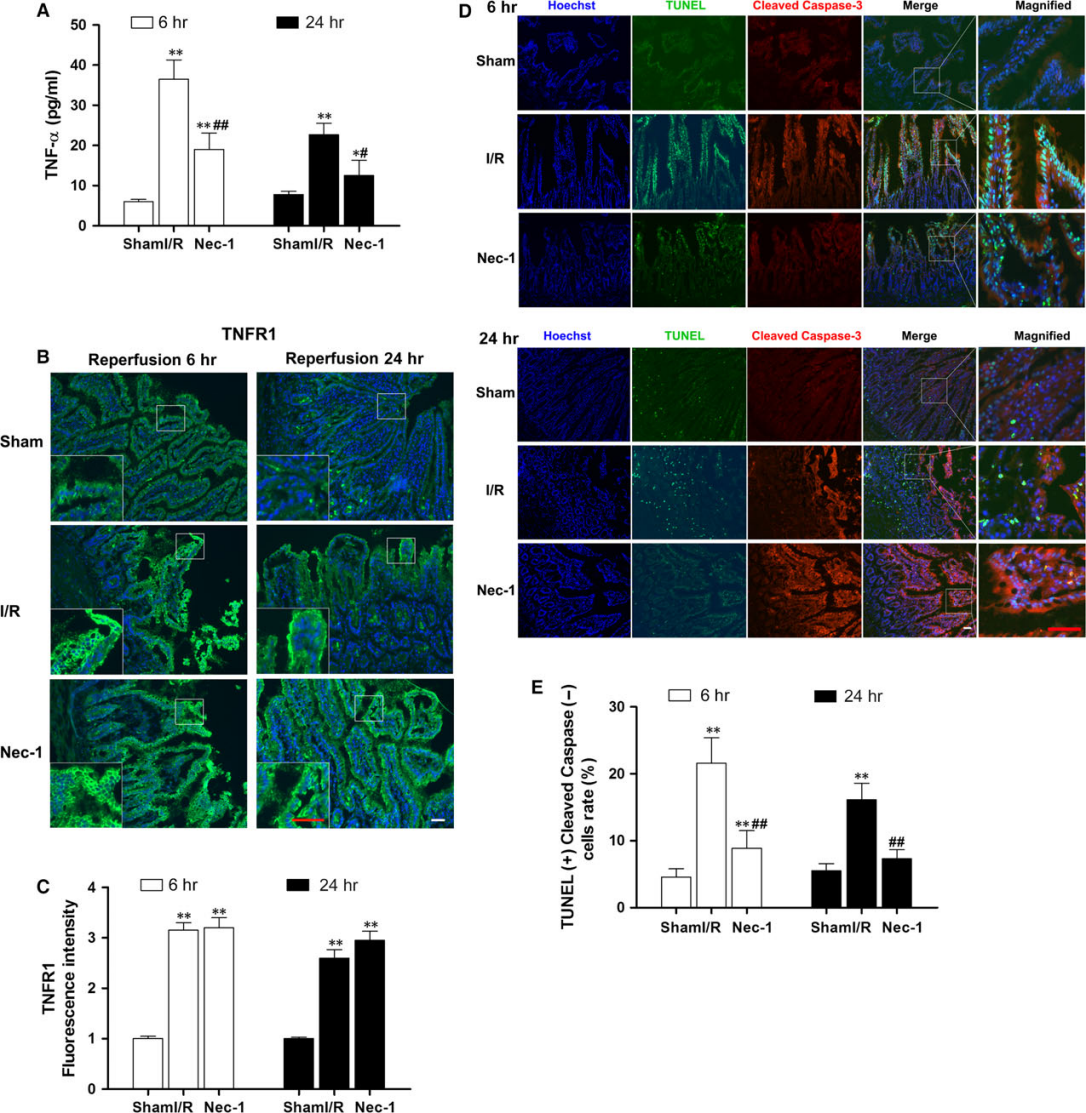
Necroptosis is an essential contributor to intestinal I/R injury *in vivo*. (**A**) Serum concentration of tumour necrosis factor‐α (TNF‐α). (**B**) Expression of tumour necrosis factor receptor 1 (TNFR1) (green fluorescence). Nuclei were counterstained with Hoechst (blue). (**C**) Fluorescent intensity of TNFR1 in the intestine. (**D**) TUNEL and cleaved caspase‐3 dual immunofluorescent labelling in the intestine. TUNEL (green) and cleaved caspase‐3 (red) staining were performed after 6 and 24 hrs of reperfusion. Nuclei were stained with Hoechst (blue). Both TUNEL‐ and cleaved caspase‐3‐positive cells were apoptotic, while TUNEL‐positive but cleaved caspase‐3‐negative cells were necroptic. (**E**) The number of TUNEL(+)/cleaved caspase‐3(‐) cells per ×20 field in the intestine. Bar denotes 40 μm; inset, magnified photographs. The data are shown as the means ± S.D. (*n* = 8 per group). *P < 0.05, **P < 0.01 compared with sham group; ^#^ *P* < 0.05, ^##^ *P* < 0.01 compared with I/R group.

As seen in Figure 2A, the serum TNF‐α levels in rats were clearly increased 6 and 24 hrs after reperfusion (all *P* < 0.01 *versus* sham group), whereas Nec‐1 administration before ischaemia provided salutary effects on TNF‐α release, especially after 6 hrs of reperfusion (nearly a 50% reduction). In response to the augmented TNF‐α levels, TNFR1 expression was increased ranged from trace‐detectable in the sham rats to well‐observed after 6 and 24 hrs of reperfusion in I/R and Nec‐1 groups (all *P* < 0.01 *versus* sham group, Fig. 2B and C).

Double labelling with TUNEL and cleaved caspase‐3 immunofluorescence was performed after 6 and 24 hrs of reperfusion (Fig. 2D). Both TUNEL‐ and cleaved caspase‐3‐positive (+) cells were defined as apoptotic, while TUNEL‐positive (+) but cleaved caspase‐3‐negative (−) cells were known as necroptotic. The analysis showed that Nec‐1 pre‐treatment significantly reduced the number of TUNEL(+)/cleaved caspase‐3(−) cells after both 6 and 24 hrs of reperfusion (all *P* < 0.01 *versus* I/R group, Fig. 2E).

### Necrostatin‐1 reduces necroptosis‐related protein expression and protects the intestine independent of apoptosis

**Figure 3:**
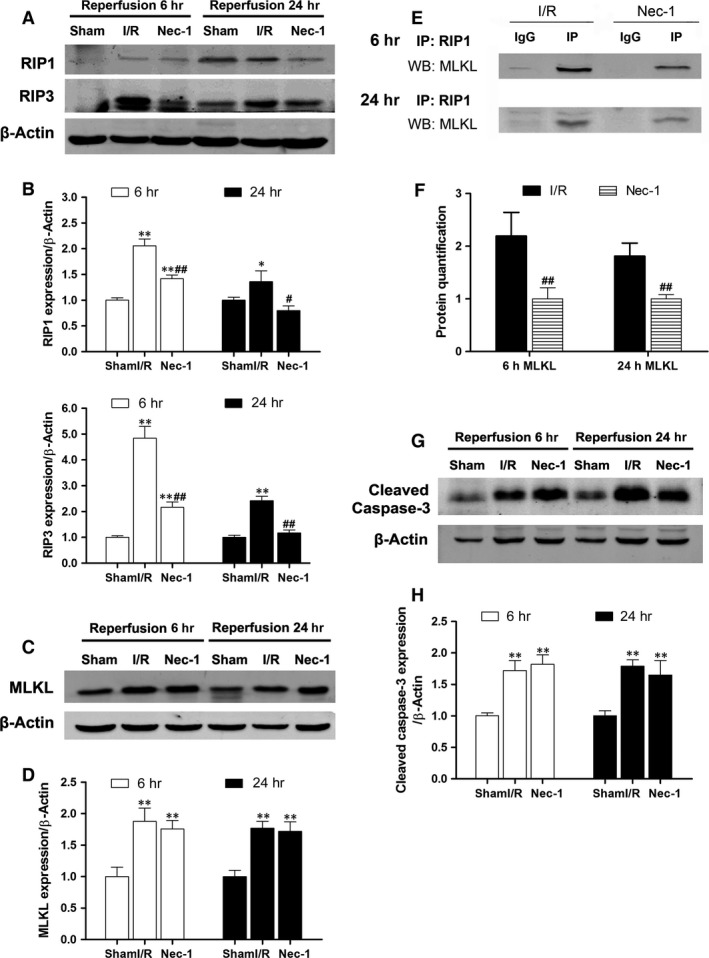
Necrostatin‐1 inhibits necroptosis‐related protein expressions and protects the intestine independent of apoptosis *in vivo*. (**A** and **B**) Western blot and quantification showed increased RIP1 and RIP3 protein expression levels after intestinal I/R. (**C** and **D**) Western blot and quantification showed increased MLKL protein expression levels after intestinal I/R. (**E** and **F**) MLKL recruitment to RIP1 was significantly decreased after Nec‐1 treatment. (**G** and **H**) Pre‐treatment with Nec‐1 did not affect caspase‐3 cleavage. The data are shown as the means ± S.D. (*n* = 8 per group). *P < 0.05, **P < 0.01 compared with sham group; ^#^ *P* < 0.05, ^##^ *P* < 0.01 compared with I/R group.

Both RIP1 and RIP3 were significantly increased after I/R‐treatment compared to sham animals both at 6 and 24 hrs of reperfusion (Fig. 3A and B). Receptor‐interacting protein 3 expression was markedly increased in I/R‐treated animals, but not after Nec‐1 treatment. Meanwhile, MLKL protein expression was significantly increased after intestinal I/R insult (Fig. 3C and D). Interestingly, Nec‐1 treatment markedly reduced the amount of MLKL protein recruited to RIP1 (Fig. 3E and F). Meanwhile, the expression of cleaved caspase‐3 was significantly increased after intestinal I/R compared to the sham groups (all *P* < 0.01). Treatment with Nec‐1, however, did not affect the level of cleaved caspase‐3 activation (Fig. 3G and H).

### Combined blockade of necroptosis and apoptosis confers better protection against intestinal I/R injury and the two pathways can be converted to one another when one is inhibited *in vivo*

**Figure 4:**
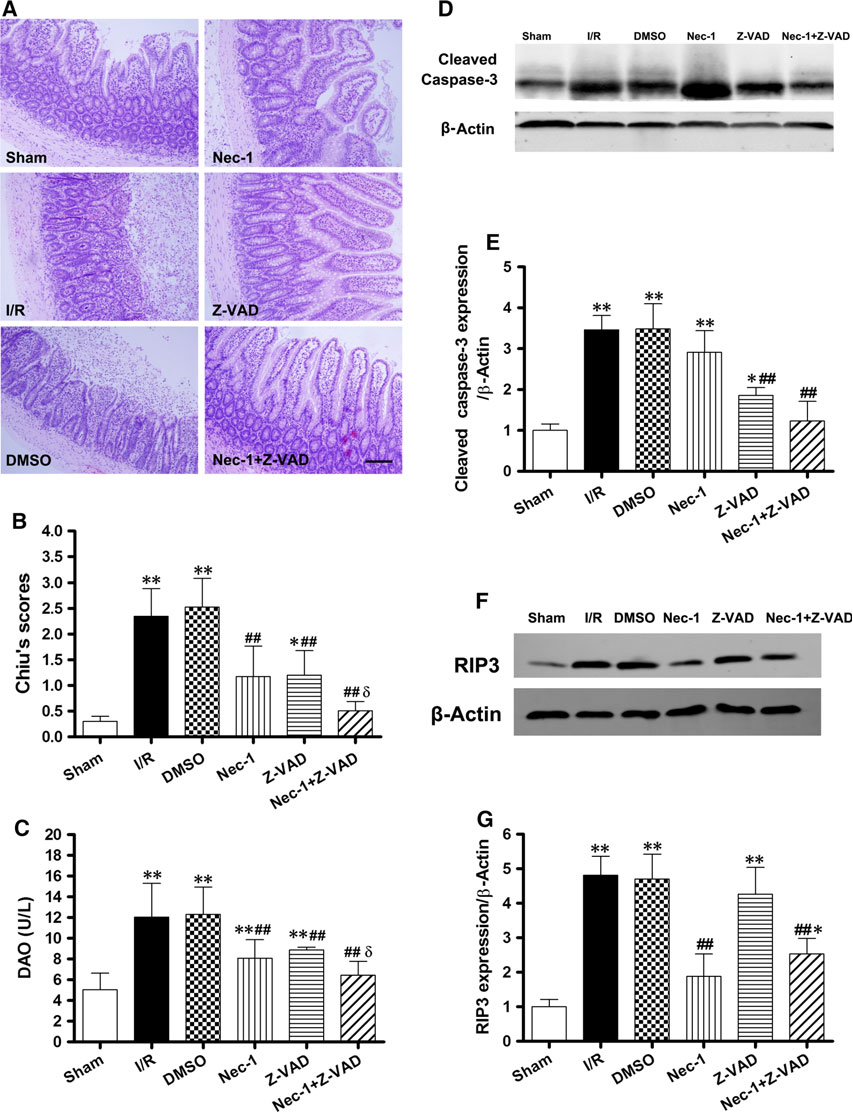
Increased protection from intestinal I/R injury by the combined blockade of necroptosis and apoptosis after 1 hr of ischaemia/24 hrs of reperfusion *in vivo*. (**A**) Histopathologic changes of the intestinal mucosa. Haematoxylin and eosin stained small intestine. Magnification is ×200, bar denotes 100 mm. (**B**) Injury scores of the intestinal mucosa morphology. (**C**) Intestinal cellular injury was evaluated by serum DAO activity. (**D** and **E**) Z‐VAD with/without Nec‐1 treatment decreased the caspase‐3 cleavage. (**F** and **G**) Treatment with Z‐VAD alone had no effect on RIP3 up‐regulation. Caspase inhibition shifted intestinal I/R‐induced epithelial cell death from apoptosis to necroptosis. The images are representative for each group. The data are shown as the means ± S.D. (*n* = 8 per group). *P < 0.05, **P < 0.01 compared with sham group, ^##^ *P* < 0.01 compared with I/R group and DMSO group, ^δ^ *P* < 0.05 compared with Nec‐1 group and Z‐VAD group.

As seen in Figure 4A, Nec‐1 and Z‐VAD independently largely abrogated the intestinal mucosal injury after 24 hrs of reperfusion, confirmed by a significant reduction in the intestinal tissue damage scores (Fig. 4B). Moreover, the combined treatment of Nec‐1 and Z‐VAD further reduced the mucosal injury. In parallel with the mucosal morphologic changes, DAO activities in the Nec‐1 + Z‐VAD group were significantly lower than the other groups (*P* < 0.01 or 0.05, Fig. 4C).

Meanwhile, the levels of cleaved caspase‐3 were increased in the I/R, DMSO, and Nec‐1 groups after 24 hrs of reperfusion. Z‐VAD with/without Nec‐1 treatment decreased the expression of cleaved caspase‐3 (Fig. 4D and E). Conversely, Z‐VAD did not affect RIP3 up‐regulation after 24 hrs of reperfusion, whereas Nec‐1 administration decreased the level of RIP3 in the Z‐VAD group (Fig. 4F and G).

### Necrostatin‐1 decreases IEC‐6 cell death and pro‐inflammatory cytokine gene expression after OGD

**Figure 5:**
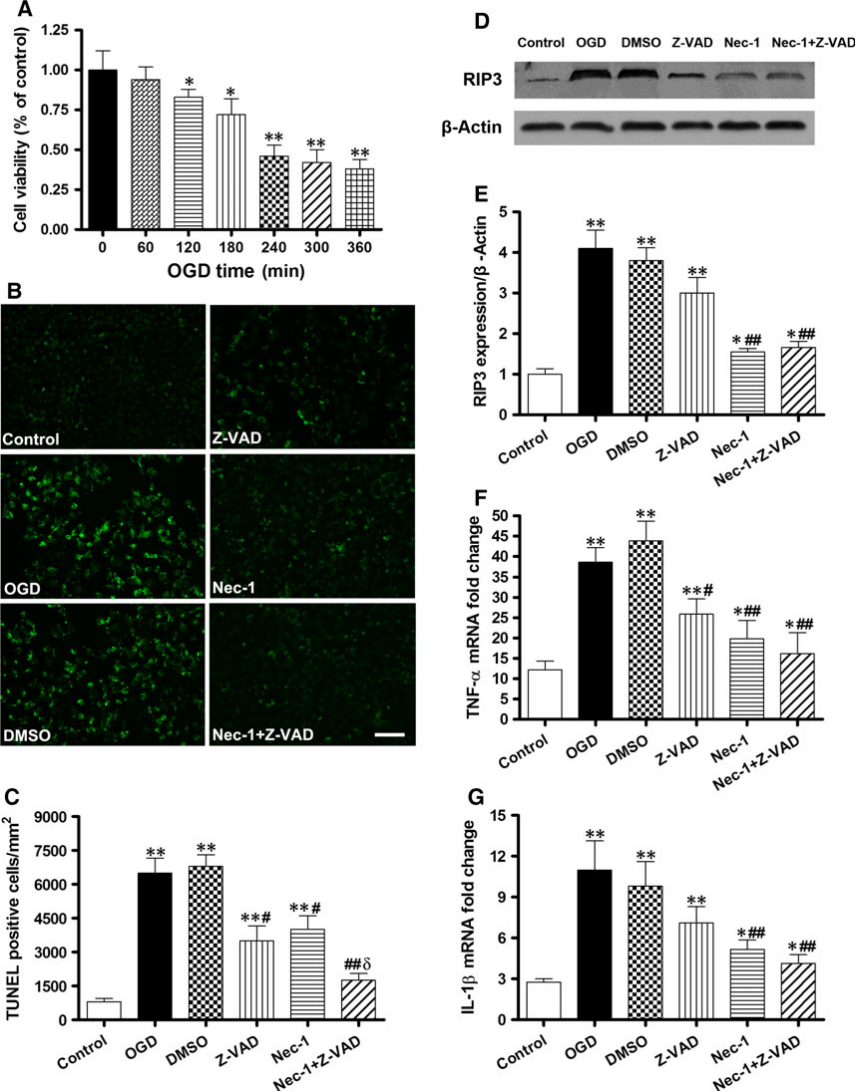
Necrostatin‐1 decreases IEC‐6 cell death and pro‐inflammatory cytokine gene expression after OGD *in vitro*. Cultured IEC‐6 cell injury was induced by depriving culture media of oxygen and glucose (OGD). (**A**) Viability after different time courses of OGD. (**B**) Immunofluorescence for TUNEL staining (green, bar denotes 20 μm) and the quantification of TUNEL‐positive cells per ×20 field in IEC‐6 cells (**C**). (**D** and **E**) Western blot and quantification show that RIP3 proteins were expressed at a higher level in the OGD, DMSO and Z‐VAD groups but were attenuated after Nec‐1 treatment. (**F** and **G**) Q‐PCR for TNF‐α and IL‐1β mRNA levels in OGD‐challenged IEC‐6 cells after Z‐VAD and Nec‐1 treatment. The data are shown as the means ± S.D. (*n* = 6 per group). *P < 0.05, **P < 0.01 compared with control group, ^#^ *P* < 0.05, ^##^ *P* < 0.01 compared with OGD group and DMSO group, ^δ^ *P* < 0.05 compared with Z‐VAD group and Nec‐1 group.

Since the OGD challenge consists of combining the deprivation of both oxygen and glucose, thereby mimicking the lack of blood supply that occurs during ischaemia in tissue. Cell viability analysis using the MTT assay showed a time‐dependent induction of injury with marked cell death (nearly a 60% reduction in viability, Fig. 5A) occurring after 240 min. of OGD. This model was used in subsequent experiments.

Treatment of Nec‐1 or Z‐VAD alone showed significant reductions in the number of TUNEL (+) cells compared with the OGD and DMSO groups (*P* < 0.05, Fig. 5B and C). Moreover, the combined treatment of Nec‐1 and Z‐VAD markedly suppressed the number of TUNEL (+) cells in comparison to the other treated groups (*P* < 0.01 or 0.05). Meanwhile, RIP3 protein was expressed at a higher level in the OGD, DMSO and Z‐VAD groups but at a lower level after Nec‐1 treatment (Fig. 5D and E).

In accordance with the reduction in RIP3 expression, Nec‐1 pre‐treatment significantly inhibited TNF‐α and IL‐1β mRNA levels after 24 hrs of reoxygenation compared with the OGD and DMSO groups (*P* < 0.01, Fig. 5F and G).

### Necrostatin‐1 confers IEC‐6 protection against OGD challenged *in vitro via* inhibiting HMGB1‐TLR4/RAGE signalling activation

**Figure 6:**
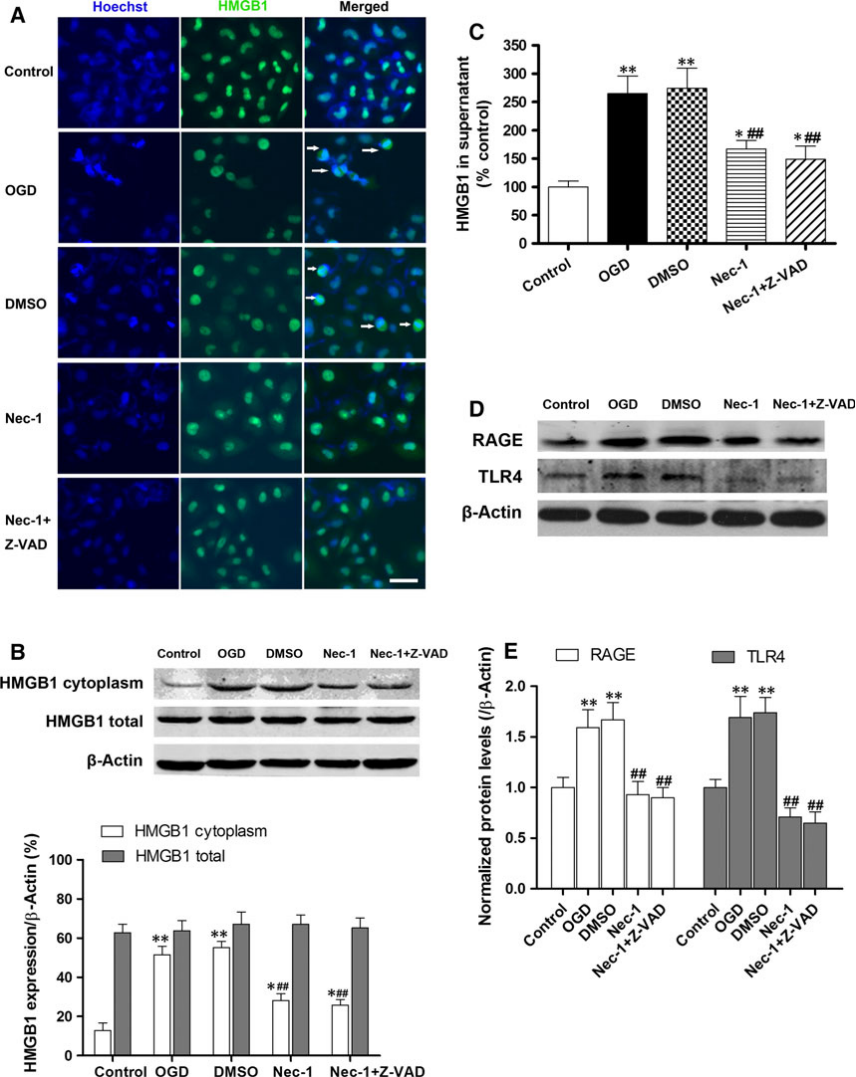
Necrostatin‐1 inhibits OGD‐induced HMGB1 translocation from the nucleus to the cytoplasm and HMGB1 signalling activation. (**A**) Translocation of HMGB1 (green) was detected in cells after OGD challenge. The nuclei were stained with Hoechst (blue, bar denotes 20 μm). White arrows indicate HMGB1 translocation from the nucleus to the cytoplasm. (**B**) IEC‐6 cytoplasmic and total extracts were analysed for HMGB1 expression by Western blot. (**C**) Supernatants HMGB1 levels were analysed by ELISA. (**D** and **E**) Western blot and quantification show the down‐regulated expression of RAGE and TLR4 after Nec‐1 pre‐treatment. The data are shown as the means ± S.D. (*n* = 6 per group). *P < 0.05, **P < 0.01 compared with control group, ^##^ *P* < 0.01 compared with OGD group and DMSO group.

The nuclear localization of HMGB1 was observed in the sham IEC‐6 group (Fig. 6A). Following OGD challenged, however, cytoplasmic HMGB1 was significantly increased in IEC‐6 cells (white arrow). Meanwhile, cytoplasmic HMGB1 was increased after OGD, suggesting active HMGB1 release (Fig. 6B). However, total HMGB1 protein levels were not significantly changed. Nec‐1 administration significantly attenuated the rise of cytoplasmic HMGB1 expression (*P* < 0.01 *versus* the OGD and DMSO groups). Furthermore, OGD injury led to clear HMGB1 release into the media which could be reduced by Nec‐1 pre‐treatment (Fig. 6C). Both RAGE and TLR4 expression levels were markedly increased in IEC‐6 cells after OGD challenge (Fig. 6D and E). However, pre‐treatment with Nec‐1 resulted in dramatic decreases in TLR4 and RAGE expression to levels even lower than that of the control.

## Discussion

In this study, our findings provide evidence that necroptosis is of important functional relevance to intestinal I/R injury *in vivo* and in OGD‐challenged IEC‐6 injury *in vitro*. Simultaneous blockade of necroptosis and apoptosis using Nec‐1 and Z‐VAD conferred enhanced intestinal protection, and the underlying mechanisms seem to be attributable to the inhibition of RIP1/RIP3/MLKL complex formation and subsequent HMGB1 signalling.

Although key regulating proteins for epithelial apoptosis and anti‐apoptotic signalling have been extensively studied during the process of intestinal I/R 6, 8, 32, necrosis is just interpreted as ‘good given’ and therefore beyond therapeutic intervention. However, there has revealed the existence of a necrotic signalling pathway leading to cell death 33. Necroptosis is morphologically characterized as necrotic cell death and is mediated by the kinases RIP1 and RIP3, both of which form a necroptosis‐inducing protein complex. Moreover, the activation of death receptors could induce cell death by executing alternative cell death pathways, such as apoptosis or necroptosis 34. But the interactions of apoptosis and necroptosis remain unexplored. Our present results show that a single administration of Nec‐1 before ischaemia markedly attenuated intestinal mucosal injury after reperfusion, as demonstrated by significantly restored in villus height and architecture and a decrease in Chiu's score (Fig. 1). The simultaneous application of Nec‐1 and Z‐VAD had a significant effect on intestinal epithelial cell protection, compared with Nec‐1 or Z‐VAD treatment alone *in vivo* and *in vitro*. We also showed that pre‐treatment with Nec‐1 exerted no effect on caspase‐3 cleavage, and pre‐treatment with Z‐VAD did not reduce RIP3 expression in ischaemic intestines and OGD cells. Therefore, the Nec‐1‐mediated inhibition of necroptosis is partly responsible for protecting intestinal epithelial cell death.

Although the detection of DNA fragmentation by TUNEL is used as a marker of apoptosis, it has been reported that necrosis, programmed or otherwise, also yields DNA fragments that react with TUNEL *in vivo*, rendering it difficult to discriminate between apoptosis and necrosis 35. As expected, the number of necroptotic cells was significantly suppressed after Nec‐1 treatment in our study (Fig. 2D and E). Furthermore, Nec‐1 treatment alone had no effect on the caspase‐3 activation after intestinal I/R insult (Fig. 3G and H). With the presence of Z‐VAD, a cell‐permeable pan‐caspase inhibitor, the intestinal histopathology revealed maximal protection upon cotreatment with Nec‐1 (Fig. 4A and B) and prevention of the associated increase in serum DAO levels (Fig. 4C). Meanwhile, when compared to I/R alone, the addition of Z‐VAD alone in I/R rats did not affect the expression of RIP3, which is the key kinase in promoting necroptotic cell death 36. Accordingly, it could be speculated that the necroptotic epithelial cell death is likely to account for the mucosal injury after caspase inhibition. Therefore, these findings clearly demonstrate that necroptosis is an important cause of intestinal epithelial cell death after intestinal I/R. Moreover, necroptosis may function as an essential and alternative/complementary mechanism of intestinal epithelial cell death, which was further revealed upon inhibition of apoptosis pathway.

Available researches confirm that the binding of TNF‐α to TNFR1 is one of the best‐studied extracellular signalling pathways of necroptosis induction 17, 37. After intestinal I/R, serum TNF‐α was significantly increased (Fig. 2A), and treatment with neutralizing anti‐TNF‐α antibody or inhibitor suppressed mucosal cell death 38, suggesting that TNF‐α may contribute to the induction of apoptosis and programmed necrosis. In response to augmented TNF‐α levels, TNFR1 expression was also found to be up‐regulated in the intestinal mucosal (Fig. 2B and C). It seems that Nec‐1 administration has no effect on the expression of TNFR1, and the most likely reason for this is that Nec‐1 just targets on the necrosome formation and other downstream signals (including MLKL, reactive oxygen species production, *etc*.) of TNF‐α/TNFR1‐elicited pathways. The intracellular domain of the TNFR assembles necrosis‐specific proteins, including RIP1 and RIP3. In addition, RIP3 controls necroptosis by initiating the pro‐necrotic kinase cascade and assembling of the RIP1/RIP3 complex, thus leading to the regulation of programmed necrosis 39. Mixed lineage kinase domain‐like protein, the direct substrate of RIP3, is essential for the TNFR‐mediated formation of necrosome and targeting appropriate downstream effectors in the necroptosis‐inducing process 40, 41, 42. In our current study, RIP1 and RIP3 expression were increased in the intestinal response to I/R (Fig. 2A–C). The findings that Nec‐1 administration decreased RIP1/RIP3 expression *in vivo* after intestinal I/R, together with its beneficial effect on reducing the amount of MLKL protein recruited to RIP1, suggesting that inhibition of RIP1/RIP3 expression and the interaction with MLKL are clearly involved in Nec‐1 induced protection *in vivo*, which is novel and provides additional mechanistic insights.

Oxygen‐glucose deprivation served as an *in vitro* model of intestinal I/R injury that is more amenable to the molecular dissection of cell death mechanisms. Consistent with evidence from intestinal protection *in vivo*, our *in vitro* data show that applying Nec‐1 or Z‐VAD alone 1 hr before OGD reduced the number of TUNEL (+) cells, which was then further decreased nearly to the level of the control cells after the combined application of Nec‐1 and Z‐VAD (Fig. 5B and C). The determinant role of RIP3 in necroptosis has already been demonstrated by complete abrogation of necroptotic cell death in RIP3‐depleted models 37, 43; therefore, our *in vitro* results highlight the crucial impact of RIP3 protein levels on OGD‐induced IEC‐6 necroptosis.

High‐mobility group box‐1, a highly conserved ubiquitous protein present in the nucleus and cytoplasm of cells, is known to be actively secreted by immunostimulated macrophages 44 and enterocytes 45 and is released from necrotic cells but not apoptotic cells 46. Once in the extracellular milieu, HMGB1 triggers cellular and biological inflammatory responses, and activates innate and adaptive immunity by interacting with multiple cell surface receptors 47, including RAGE and TLR2, TLR4 and TLR9 48, 49, 50. Recent studies have demonstrated that HMGB1 concentrations in circulation elevate early after intestinal I/R 51 and haemorrhagic shock 52, and HMGB1 neutralization greatly inhibits the generation of pro‐inflammatory cytokines (IL‐6, TNF‐α and NF‐κB p65) 53, ameliorates mucosal barrier dysfunction and improves animal survival. In this study, our results show that OGD induces HMGB1 translocation from the nucleus to the cytoplasm (as demonstrated by immunofluorescence and increased cytoplasmic HMGB1 expression) and is released into the supernatant, which suggests that the active secretion of HMGB1, in addition to its passive release from damaged cells is Nec‐1 dependent. Furthermore, pre‐treatment of Nec‐1 resulted in markedly attenuated of TLR‐4 and RAGE expression associated with decreased IEC‐6 cell death. We propose that Nec‐1 may prevent increased expressions of TLR‐4 and RAGE by attenuating IEC‐6 necroptosis (as demonstrated by lower RIP3 expression and reduced TUNEL staining). We also further explored the well‐described anti‐inflammatory effects of Nec‐1 (reduced serum TNF‐α levels, TNF‐α and IL‐1β gene expression). These results were consistent with previous reports in other disease models in which Nec‐1 inhibits cellular neuroinflammation after controlled cortical impact 54 and reduces leucocyte/macrophage influx and the expression of oxidative stress genes after myocardial I/R 19. Therefore, it is plausible that the anti‐inflammatory actions of Nec‐1 contribute to the reduced TLR‐4 and RAGE expression levels following OGD challenge.

There were several limitations in our experimental design. First, we pre‐treated Nec‐1 30 min. before intestinal ischaemia. It is unlikely that Nec‐1 pre‐treatment could be applied at this time‐point in clinical practice, which may limit the clinical use of Nec‐1. Further studies are needed to determine practical and effective regimens of Nec‐1 after intestinal ischaemia. Second, we used a single dose of Nec‐1 in this study. In a future study, multiple Nec‐1 doses should be evaluated to confirm the optimal dose and therapeutic window of Nec‐1 for maximal intestinal protection. Third, given the complexity of bacterial flora and diversity of cell population (enterocytes, Panth cells, goblet cell, *etc*.) with highly specialised functions in the intestine, it is difficult to clearly elucidate the molecular dissection of cell death mechanisms only by using an *in vivo* system. In addition, the parallel existence of necroptotic and apoptotic pathways that induce cell death in the same organ following the same ischaemic stimulus demonstrates the complexity in the pathophysiology of intestinal I/R and the regulation process requires further exploration.

**Figure 7:**
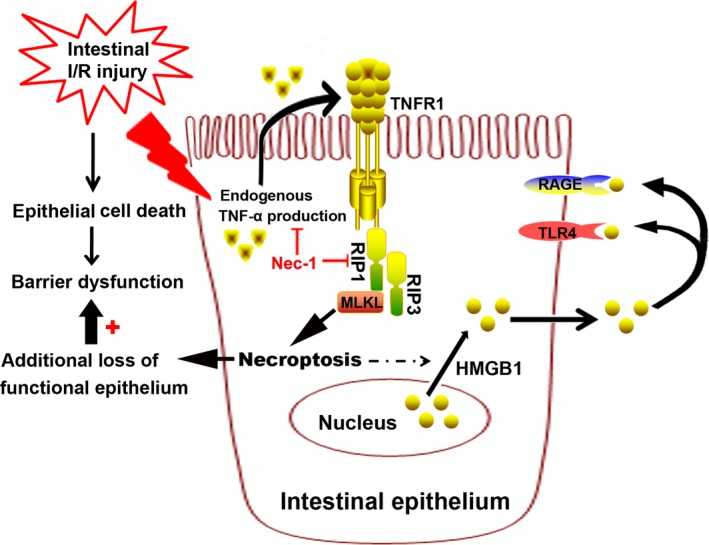
Putative mechanism of necroptosis in the development of intestinal injury following intestinal I/R. In the proposed model, intestinal I/R stimulates the endogenous TNF‐α production and TNFR1 combination, and activation of the downstream RIP1/RIP3‐MLKL signalling pathway, which triggers necroptotic intestinal epithelium death and release of HMGB1 from the nucleus to the cytoplasm, further contributing to the additional loss of functional epithelium and subsequent mucosal barrier dysfunction. Nec‐1 administration reduced the number of necroptotic cells and extracellular HMGB1 level, which decreases the inflammatory drive that both increases TLR4 and RAGE expression and activates the detrimental effect of HMGB1 signalling in intestinal ischaemia.

In summary, upon increased endogenous TNF‐α binds to the TNFR1 after intestinal I/R, the receptor becomes activated and recruits a complex of proteins to trigger RIP‐mediated necroptosis (summarize in Fig. 7). Our results suggest that pre‐treatment with the specific inhibitor Nec‐1 reduces necroptosis *via* the downregulation of RIP1/RIP3‐MLKL recruitment and the inhibition of downstream HMGB1 signalling, at least in IEC‐6 cells *in vitro*, and that Z‐VAD cotreatment provides effective synergistic protection. These finding might promise new therapeutic possibilities for the treatment of equivalent human pathologies in the future.

## Conflict of interest

The authors confirm that there are no conflicts of interest.
